# microRNA-128a dysregulation in transgenic Huntington’s disease monkeys

**DOI:** 10.1186/1756-6606-7-46

**Published:** 2014-06-13

**Authors:** Jannet Kocerha, Yan Xu, Melinda S Prucha, Dongming Zhao, Anthony WS Chan

**Affiliations:** 1 Division of Neuropharmacology and Neurologic Disease, Yerkes National Primate Research Center, 954 Gatewood Rd., N.E Atlanta, GA 30329, USA; 2 Department of Human Genetics, Emory University School of Medicine, 615 Michael St., Whitehead Building, Atlanta, GA 30322, USA; 3 Current address: Department of Chemistry, Georgia Southern University, Statesboro, GA 30458, USA

**Keywords:** microRNAs, Noncoding RNAs, Huntington’s disease, Brain, miR-128a

## Abstract

**Background:**

Huntington’s Disease (HD) is a progressive neurodegenerative disorder with a single causal mutation in the Huntingtin *(HTT)* gene. MicroRNAs (miRNAs) have recently been implicated as epigenetic regulators of neurological disorders, however, their role in HD pathogenesis is not well defined. Here we study transgenic HD monkeys (HD monkeys) to examine miRNA dysregulation in a primate model of the disease.

**Results:**

In this report, 11 miRNAs were found to be significantly associated (P value < 0.05) with HD in the frontal cortex of the HD monkeys. We further focused on one of those candidates, miR-128a, due to the corresponding disruption in humans and mice with HD as well as its intriguing lists of gene targets. miR-128a was downregulated in our HD monkey model by the time of birth. We then confirmed that miR-128a was also downregulated in the brains of pre-symptomatic and post-symptomatic HD patients. Additionally, our studies confirmed a panel of canonical HD signaling genes regulated by miR-128a, including *HTT* and Huntingtin Interaction Protein 1 (*HIP1*).

**Conclusion:**

Our studies found that miR-128a may play a critical role in HD and could be a viable candidate as a therapeutic or biomarker of the disease.

## Background

Over the past decade, the microRNA (miRNA) class of noncoding RNAs (ncRNAs) has consistently been implicated in the neurogenesis, neurodegenerative, and synaptic plasticity functions of the central nervous system (CNS) [1-9]. Modulation of miRNA activity has been associated with a broad scope of CNS disorders, including schizophrenia, autism, Fragile X, addiction, Parkinson’s Disease (PD), Alzheimer’s Disease (AD), Frontotemporal Dementia (FTD), and Huntington’s Disease (HD) [10-17]. Indeed, the role of miRNA dysregulation in neurological diseases has come under increasingly intense study during recent years in the quest for novel therapeutic strategies [9,18].

HD is characterized by progressive brain atrophy, particularly in the striatum and hippocampus, with associated deficits in cognitive, behavioral, and motor functions [19]. Although the causative factor for HD is monogenic and results from expansion in the polyglutamine (polyQ) region of the Huntingtin (*HTT*) gene, there is wide variability in age of onset, polyQ length, and degree of symptoms [20,21]. Published reports indicate that epigenetic factors, such as ncRNA regulation, work in union with the genetic anomalies to provoke pathogenic outcomes in HD [22-28].

MiRNA dysregulation has been reported in HD patients, transgenic HD mice, and *in vitro* experimental models [22-28]. Here we examine the miRNA expression profile of fetal and newborn frontal cortex from HD monkeys, with corresponding neuropathological analysis from a subset of those monkeys. The HD monkeys examined in this study carry the exon 1 of human *HTT* gene with an expanded polyQ tract of 27Q – 122Q (Additional file 1: Table S1), a range well above the polyQ repeat of 10-11Q in wild-type (WT) rhesus macaque [29,30]. We identified a significant dysregulation of 11 miRNAs in the HD monkeys and a correlation of their gene targets with the HD canonical signaling pathway. Interestingly, through bioinformatic analysis and subsequent *in vitro* studies, miR-128a was found to regulate the 3’-untranslated regions (3’ UTRs) of the *HTT* and Huntingtin Interacting Protein 1 (*HIP1*) genes. Additionally, analysis of previously reported datasets revealed that miR-128a was also downregulated in the brain tissue of HD mice and humans [22-25]. We expand on these datasets to show that miR-128a is downregulated in a cohort of both pre-symptomatic and post-symptomatic HD patients.

Overall, we report miRNA dysregulation in the brains of HD monkeys, with parallel miRNA dysregulation in human patients. We find that neuronally-enriched miRNAs may play a fundamental role in HD pathogenesis. Additionally, we show that the miRNAs implicated in HD may regulate a significant network of genes within the HD canonical signaling pathway, providing a new paradigm approach for a disease with complex molecular dysregulation.

## Results

### Genotyping and mRNA expression analysis of HD monkeys

**Figure 1:**
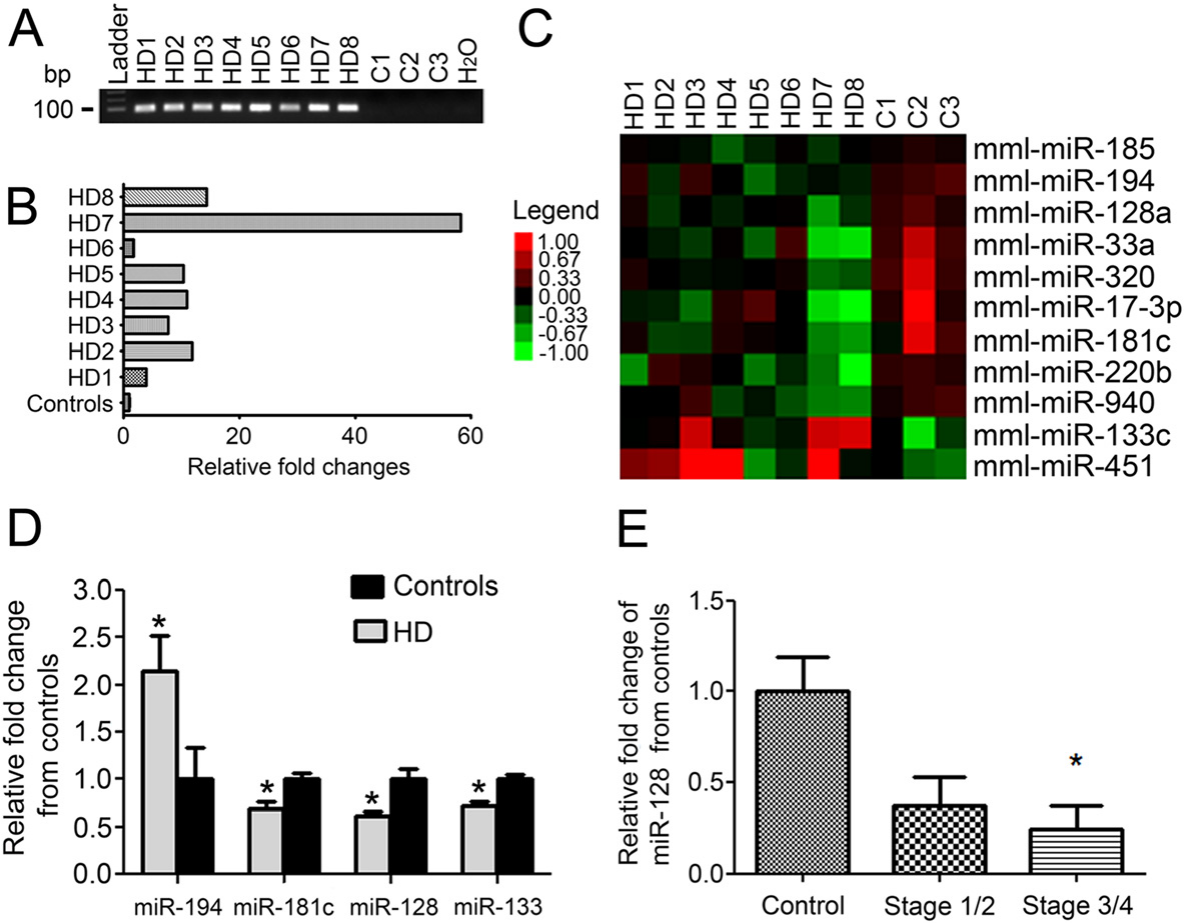
**Genotype and miRNA profiling analysis from HD monkey brains and human miR-128a expression.A)** Genomic DNA (gDNA) was isolated from the frontal cortex of 3 GFP control (C1-C3) and 8 HD monkeys (HD1-HD8). The gDNA was amplified using specific primers for *mHTT* and the PCR product was analyzed by gel electrophoresis. **B)** The fold change of *HTT* mRNA levels for the HD monkeys compared to the controls was quantitated by qPCR. **C)** Cluster diagram for dysregulated miRNAs in HD transgenic cortex. miRNA expression values (Log2 transformed & normalized microarray probe intensities) of the dysregulated microRNAs (P value < 0.05) in the HD and GFP control monkey cortex were median centered. Green squares represent lower than median levels of gene expression; black squares represent median levels of gene expression; red squares represent higher than median levels of gene expression. Legend units: 1.0 = differs from median probe intensity by one log 2 unit (2-fold). **D)** qPCR validation for the expression of 4 of the 5 miRNAs with significant Ingenuity analysis association to the HD pathway (miR-940 also showed association with the HD pathway, however, no Taqman assays were commercially available). *T*-test was used to determine significant differences between HD and control monkeys (P Value < 0.05 indicated by *). **E)** Expression of miR-128a was quantitated from control, pre-symptomatic (HD), and post-symptomatic (HD) human striatum samples. Total RNA was extracted and probed with specific Taqman primers to miR-128 for qPCR. T-tests were used to determine significant differences compared to the control group (P Value < 0.05 indicated by *).

Genomic DNA (gDNA) isolated from all the control and HD monkeys was analyzed by PCR for detection of the *mHTT* transgene. The amplification products were subjected to agarose gel electrophoresis. Amplicons of ~100 bp were observed in samples from HD monkeys but not from control monkeys (Figure 1A). These observations are consistent with the anticipated results, as the primers amplified the junction between the vector backbone and the 5’ region of the exon 1 of the mHTT transgene, which distinguish between the *mHTT* transgene and the endogenous wild-type *HTT* gene (see Additional file 2: Table S2 for primer sequences).

In addition to confirming the genotype of each HD monkey, the expression of HTT mRNA in cortical tissues was also measured. All HD monkeys displayed significantly higher levels of HTT transcript levels than the controls, with HD7 displaying the highest expression (Figure 1B). Primers for the mRNA analysis were not designed to distinguish between the mutant and wild-type *HTT* transcripts, therefore, the relative expression of *mHTT* in the HD monkeys was determined by comparing with the endogenous transcript levels in the controls.

### Expression of mHTT aggregates in HD monkey frontal cortex

**Figure 2:**
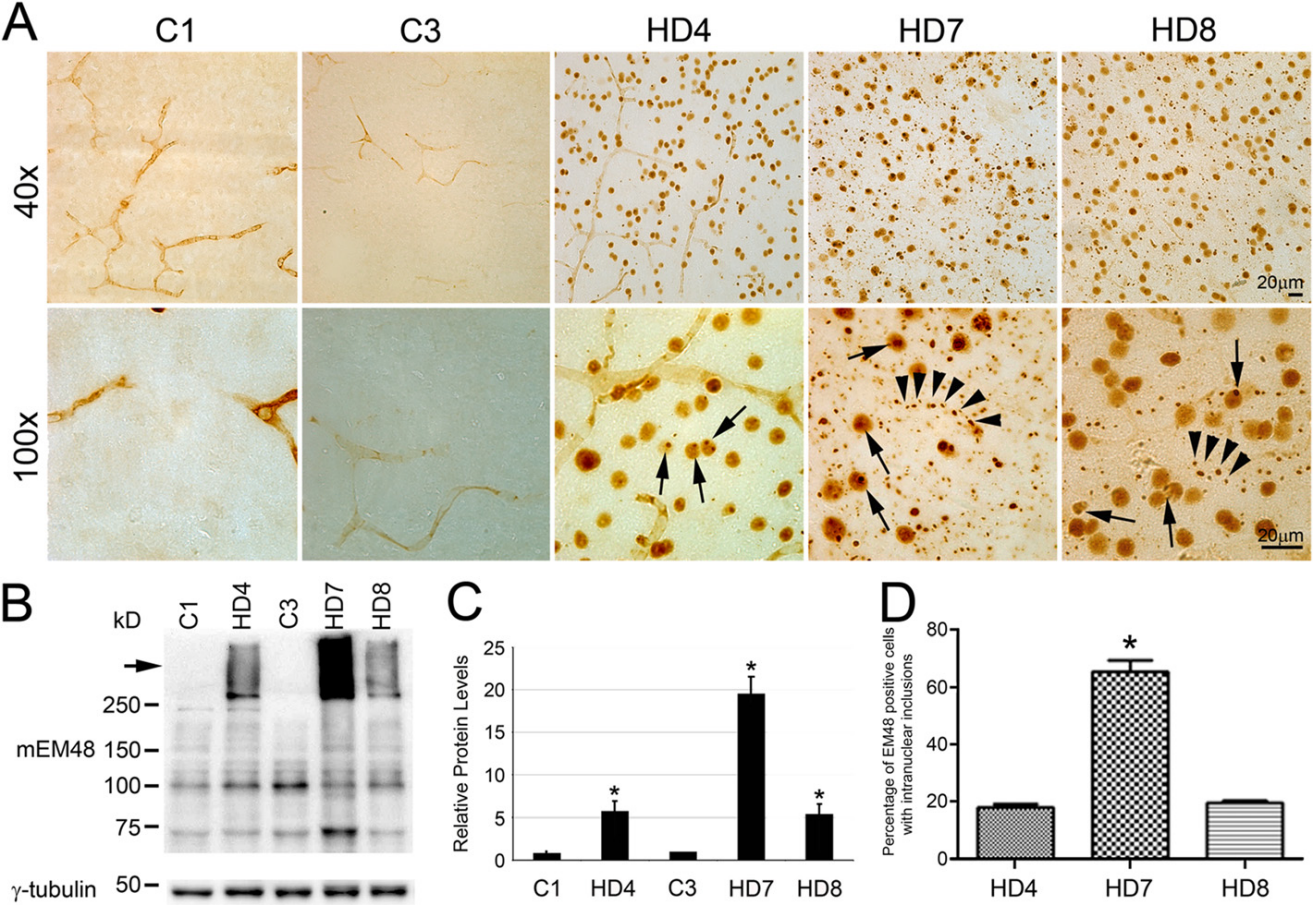
**Expression of mHTT in monkey frontal cortex. A)** Brain sections of HD monkey (rHD4, rHD7 and rHD8) and GFP monkey (C1 and C3) were immunostained with mEM48. Low magnification (upper panels, 40×) and high magnification (lower panels, 100×) shows that mHTT forms intranuclear inclusions (arrows) and neuropil aggregates (arrowheads). **B)** mEM48 immunoblot of frontal cortex showed high-molecular-mass mutant HTT aggregate (arrow) in the stacking gel. γ- tubulin was used as an internal control. **C)** Quantification of the mHTT aggregate in the stacking gel showed the relative level of mHTT aggregates in the frontal cortex. All HD monkeys have significantly higher level of mHTT aggregate compared to the controls. **D)** The percentage of mEM48 positive cells with or without intranuclear inclusions was calculated and compared among HD monkey frontal cortex immunostained with mEM48. HD7 has significantly more nuclei with intranuclear inclusions than HD4 and HD8. The data was presented as mean ± SE. *P < 0.05.

Immunohistochemistry with mEM48, an antibody that specifically recognizes mutant HTT (mHTT) with expanded polyQ, revealed a wide distribution of neural cells with mHTT aggregates in the frontal cortex of the HD monkeys (HD4, HD7 and HD8), but not in the controls (C1 and C3) (Figure 2). Intranuclear inclusions were found in the nucleus (Figure 2A, arrows) and neuropil aggregates were formed along neuronal processes for HD7 and HD8 (Figure 2A, arrow head). mHTT aggregates and nuclear inclusion in HD4 were much less prevalent with no neuropil aggregates.Consistent with the immunohistochemistry results, Western blots with mEM48 revealed the presence of oligomeric mHTT aggregates in the stacking gel for the frontal cortex of all HD monkeys, with no aggregation observed in control animals (Figure 2B; stacking gel). Quantitation by densitometry revealed a significant increase of mHTT aggregates in the frontal cortex of HD monkeys compared to control animals (P < 0.05) (Figure 2C). The number of cells with intranuclear inclusions in HD7 was significantly more than those observed in HD4 and HD8, which have similar number of cells with intranuclear inclusions (Figure 2D).

### Expression of activated caspase-3 and glial fibrillary acidic protein (GFAP) in HD monkey frontal cortex

**Figure 3:**
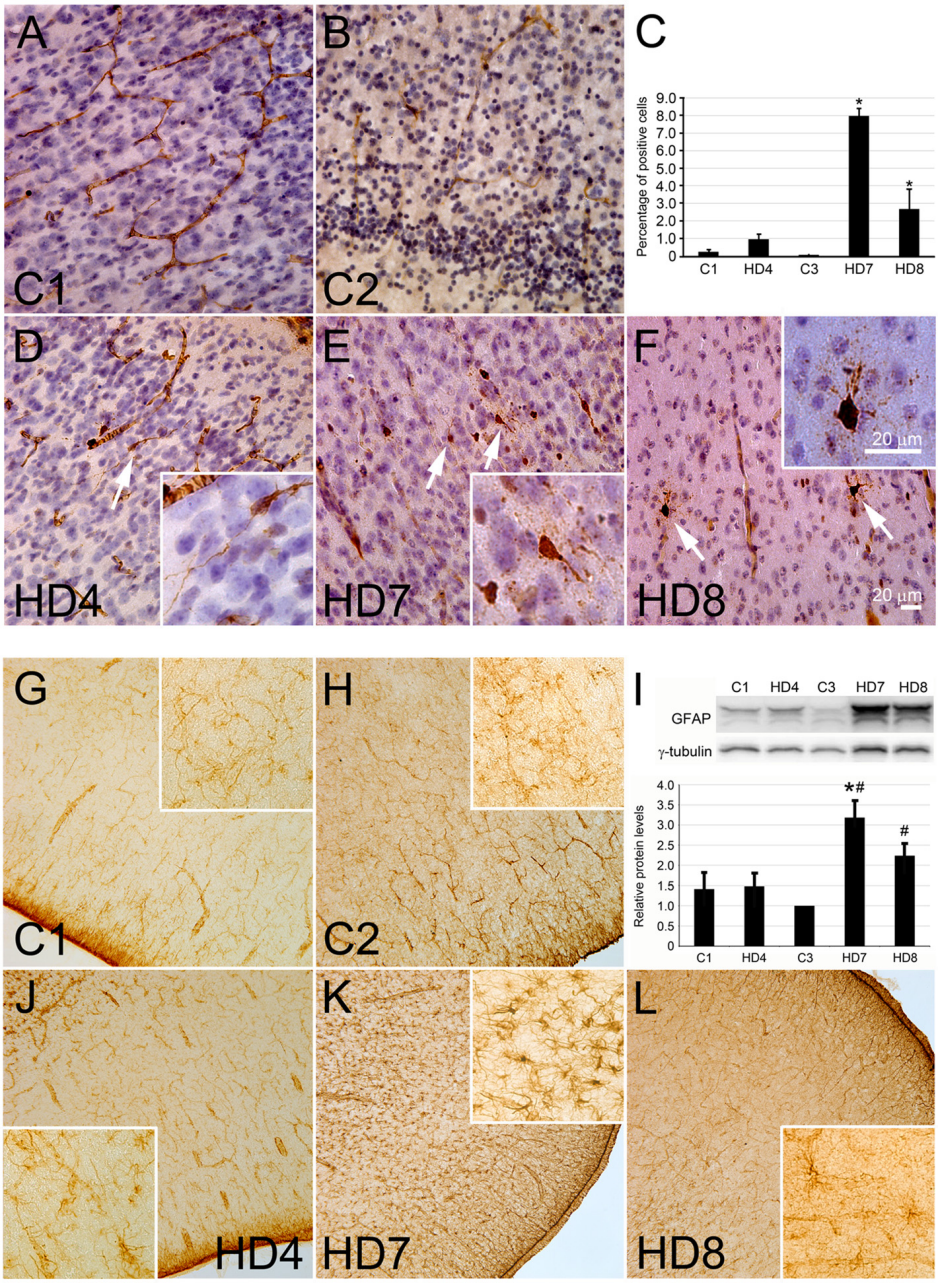
**Neuropathology in HD monkey frontal cortex. A-F)** An increased number of caspase-3 positive cells in frontal cortex of HD monkeys **(D-E)** was observed when compared with control monkeys (A-B). **C)** Quantification of caspase-3 positive cells in HD and control monkey frontal cortex showed significant difference between HD monkeys (HD7 and HD8) and control monkey (C1 or C3). However, no difference was observed between HD4 and the controls. The data are presented as mean ± SE. *P < 0.05 compared with C1, and ^#^*P* < 0.05 compared with C3. **G-L**) An increased number and intensity of GFAP positive cells was observed in HD monkeys (HD7 and HD8) but not in HD4 (J-L). High magnification (inserts) showed reactive astrocytes in HD7 and HD8 with strong GFAP staining. Quantification of GFAP expression in the HD monkey frontal cortex showed significant increase expression of GFAP in HD7 and HD8 but not in HD4. The data were presented as mean ± SE. *P < 0.05

Immunohistochemistry was performed using an antibody against the activated form of caspase-3, a signaling molecule involved in the execution phase of apoptosis. Immunostaining detected caspase-3 positive cells in the frontal cortex of HD4, HD7 and HD8; however, significantly fewer positive cells were observed in the control monkey brains (Figure 3A-F). Caspase-3 positive cells also showed enlarged cell bodies with intense nuclear staining of caspase-3 (Figure 3E and F). The number of caspase-3 positive neurons in HD7 and HD8 were significantly higher than that of the control monkeys (p < 0.05; Figure 3C). HD4 also exhibited increased numbers of caspase-3 positive neurons as compared to the control animals but was not statistically significant.GFAP immunostaining revealed increased number of astrocytic positive cells with intense staining in the frontal cortex of HD monkeys (Figure 2J-L) when compared to the controls monkeys (Figure 2G-I). Consistent with the immunohistochemical study, Western blot analysis revealed significantly increased expression of GFAP in the frontal cortex of HD7 and HD8 when compared to the control monkeys. However, GFAP level determined by western blot was not significantly different between HD4 and control monkeys (Figure 3I).

### miRNA array profiling

496 non-control transcripts (from rhesus macaque) were log_2_-transformed and normalized. Average probe intensities were filtered to generate a list of 352 rhesus miRNA probes with a fluorescence intensity exceeding threshold levels in at least 10% of the samples. Of the 352 detectable probes, 11 were significantly dysregulated in the HD monkeys with unpaired and unequal (Welch) *t*-test using Agilent GeneSpringGX 10.0 with condition P < 1 (Figure 1C and Additional file 3). Of the 11 miRNAs significantly (P < 0.05) correlated with HD pathogenesis, 9 were downregulated while 2 were upregulated compared to controls (Figure 1C).

### Target analysis of dysregulated miRNAs in HD pathogenesis

We identified the predicted mRNA targets using TargetScan for all 11 of the significantly altered miRNAs in the HD monkeys. The list of predicted mRNA targets for each miRNA was then analyzed by Ingenuity Software for pathway associations. Ingenuity analysis revealed significant pathway associations for all of the 11 miRNAs, including CREB signaling in Neurons, Chemokine Signaling, IGF-1 Signaling, and Synaptic Long Term Depression. Furthermore, when examining for shared pathways for the 11 miRNAs, we discovered that 3 pathways were associated with 7 of the miRNAs, 12 pathways associated with 6 of the miRNAs, and 13 pathways associated with 5 of the miRNAs (Additional file 4: Table S3). Notably, the HD canonical signaling pathway was associated with the mRNA targets for 5 of the 11 miRNAs.For 4 of the 5 miRNAs with significant target association to the HD canonical pathway, we confirmed their expression by qPCR in the monkey cortical tissues (miR-940 did not have commercially available Taqman primers for qPCR) (Figure 1D). For the qPCR verification, we focused on the monkeys (HD4, HD7, and HD8) that had corresponding pathology. Quantitation by qPCR revealed a significant downregulation of miR-128a in the brains of the HD monkeys (Figure 1D), consistent with our microarray results. Additionally, miR-128a was also downregulated in the brains of pre-symptomatic and post-symptomatic HD patients (Figure 1E) when compared to controls (p < 0.05).

### miR-128a regulates 3’-UTR activity of genes with known roles in HD canonical pathway

**Figure 4:**
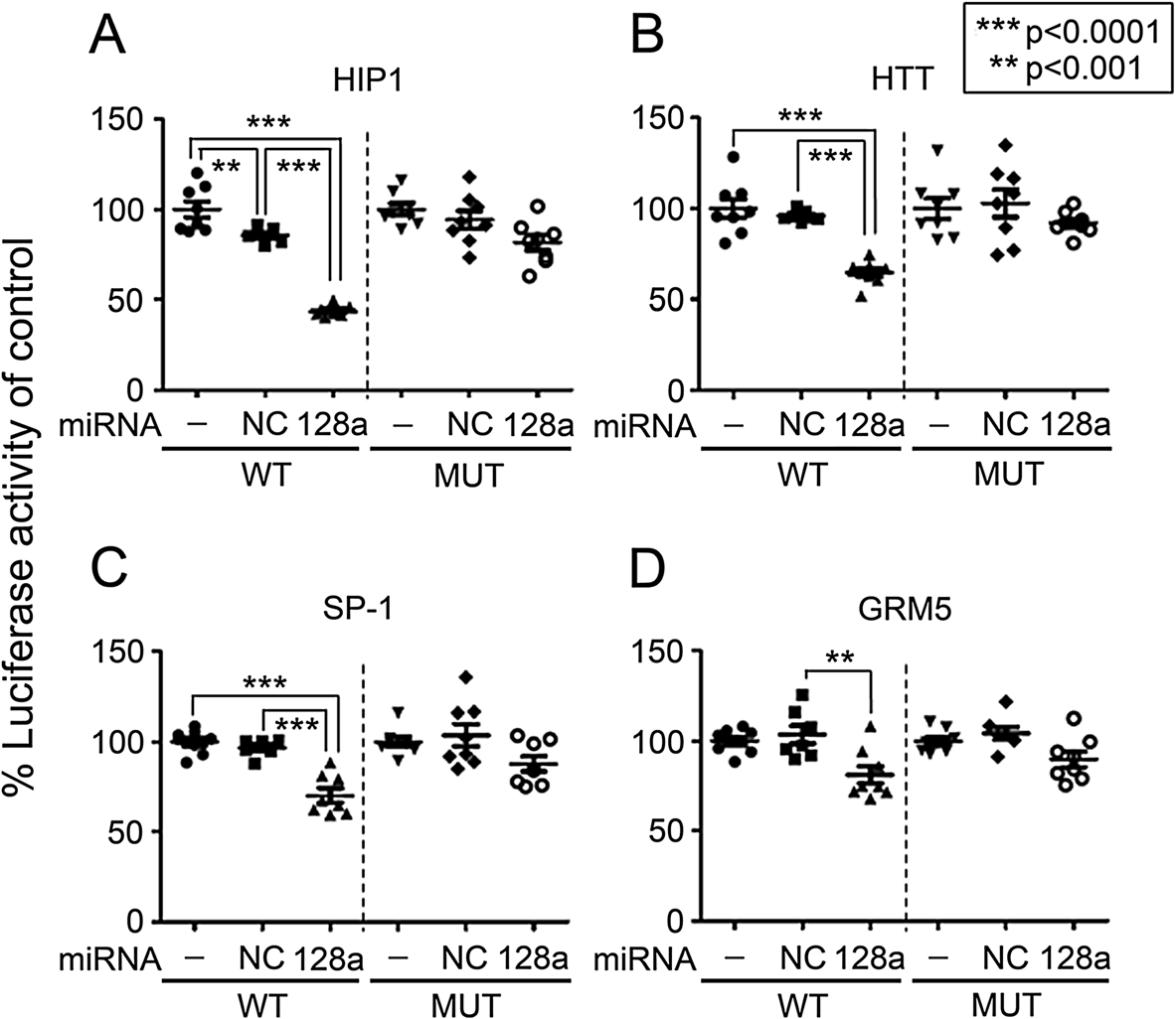
***In-vitro *analysis of HD-associated miR-128a gene targets**. The regulation of the 3’-UTR activity by miR-128 for **(A)** HIP-1, **(B)** HTT, **(C)** SP-1, and **(D)** GRM-5 was evaluated in 293-FT cells. 293-FT cells were co-transfected with 10 nM pre-miR-128a or negative control (NC) as well as luciferase reporter constructs containing the WT or mutant (MUT) 3’- predicted miR-128a binding site for each of the genes. Relative luciferase activity data were normalized as a percentage of plasmid-only control levels (100%) for each 3’-UTR construct. One-way ANOVA followed by Tukey’s post-hoc testing confirmed that miR-128a exposure resulted in significant reduction (p < 0.001) in 3’-UTR activity for each WT construct (as compared to NC); no significant differences between treatments were detected in MUT constructs.

The HD canonical signaling genes *HIP-1, HTT, SP-1, and GRM5* are all predicted gene targets of miR-128a. To determine whether miR-128a regulates the 3’-UTRs of these genes, luciferase reporter assays were developed and co-transfection assays were performed to examine the effect of miR-128a mimic on 3’-UTR activation. Transfection of miR-128a mimic significantly reduced the 3’-UTR activation of the WT constructs for HIP-1, HTT, SP-1, and GRM5 in the luciferase reporter assays (Figure 4). Suppression of HIP-1, HTT, and SP-1 by miR-128a mimic (55%, 36%, and 30% respectively) was highly significant compared to the negative control (NC) (p < 0.0001 by One-way ANOVA followed by Tukey’s post-hoc multiple comparison). GRM5 3’ UTR was suppressed by the miR-128a mimic with a significance of p < 0.001. None of the site-specific mutant (MUT) control reporter constructs for the 4 genes examined were significantly regulated by the miR-128a mimic.

## Discussion

In this study, we examine ncRNA regulation in HD monkeys and identified 11 significant disease-associated miRNAs. This is the first study which analyzes miRNA regulation in a transgenic primate model of a human disease, with a goal of helping to bridge or expand on previous results in HD rodents and human patients. The HD monkeys offer a unique resource to identify pathogenic ncRNA mechanisms that are either conserved from lower vertebrates to humans or are primate specific. For example, miR-451, one of the 11 miRNAs we found modulated in the HD monkeys, is also upregulated in HD patients [24]. However, miR-451 does not appear to be disrupted in HD mice models, suggesting the involvement of this miRNA in disease progression may be restricted to primates. On the contrary, we found miR-128a downregulated in the HD monkeys and human patients, and there are published reports also showing its corresponding downregulation in at least 2 distinct HD mice models [23]. Additionally, our human data for miR-128a corresponds with other reported datasets in HD patients [24]. The miR-128a results, however, were not previously highlighted in the previous publications.

Although it is important to identify both primate-specific and conserved molecular mechanisms in disease, we focused on miR-128a in this study due to the compelling list of genes it is predicted to target in HD, including; *SP1*, *HIP1* as well as *HTT* itself. Our in vitro luciferase reporter assays revealed the binding sites for miR-128a in *HIP1*, *SP1*, and *HTT* are all regulated by miR-128a compared to stringent site-specific mutant controls. Although we experimentally examined a subset of the predicted miR-128a gene targets, there are likely more genes which miR-128a regulates within the HD pathway. Furthermore, a few of the other 11 miRNAs we identified to be associated with HD in this study are also likely to regulate genes within the HD pathway. A future point of interest could examine whether a distinct group of miRNAs work together as a network to govern the HD genes. MiR-940, one of the 11 HD-associated miRNAs in the transgenic monkeys, is also predicted to target *HTT*. Correspondingly, although we examined miR-128a regulation of the SP1 transcription factor, other miRNAS identified in this study also bioinformatically bind to *SP1* (such as miR-320, miR-133, and miR-181). Overall, this report points to a collective miRNA targeting of *HTT* or genes which regulate *HTT* (i.e. *SP1, HIP1,* etc.*)*. Furthermore, several of the dysregulated miRNAs in the HD monkeys are also predicted to target the insulin like growth factor −1 gene (*IGF1*) or its receptor (*IGF1-R*), including; miR-128a, miR940, miR-320, and miR133. It has previously been shown that IGF1 signaling is linked to HD and other neurodegenerative diseases [31-33]. Future studies of these other miRNAs and their target genes, along with miR-128a, could help develop a more complete understanding of ncRNA regulation in HD.

miR-128a is neuronally-enriched [34,35] and one of the most abundantly expressed miRNAs in human and mouse brain [36]. A recent report indicated a principal role for miR-128a in motor activity and neuronal excitability, and its downregulation can provoke epileptic seizures [36]. HD is partly characterized by deficits in motor activity, leading to significant impact on associated movements and possible development of seizures in patients [37,38]. Tan *et al*. showed that the seizures in mice with deficient miR-128a expression can be alleviated with treatment of an anti-convulsant drug [36]. Moreover, the authors showed that the ablation of miR-128a specifically in the dopamine 1 receptor-expressing neurons (D1-neurons) leads to juvenile hyperactivity and seizures [36], consistent with other reports implicating D1-neurons in the symptoms of HD [39].

Several studies have also linked miR-128a with tumor repression and apoptosis [40,41]. Interestingly, apoptosis related signaling has consistently been implicated in HD pathogenesis [42-45]. One possibility is that the polyQ expansion in *HTT* of HD patients signals for apoptosis and neurodegenerative cascades through the regulation of miR-128a. Indeed, we found caspase-3, a component of the apoptosis pathway [44,46,47], was dysregulated in HD monkeys (Figure 2). It has also been reported that miR-128a is involved in the regulation of neuronal differentiation and survivability, including through the targeting of transcription factors and neurotrophins [48,49]. Overexpressing miR-128a represses the levels of the neurotrophin-3 receptor (*NTRK3*) gene [48,49] and the transcription factor E2F3A [50]. Moreover, it has also been shown that miR-128a can target genes involved in neuronal differentiation and viability through the regulation of nonsense-mediated decay (NMD) [28,51,52]. NMD is an RNA surveillance pathway which can provoke the degradation of a subset of RNA transcripts. It has been suggested that increased expression of miR-128a can modulate the NMD pathway, resulting in elevated levels of important neuronal proteins [28,51,52].

Overall, our results suggest that miRNAs, and more specifically miR-128a, may play a pivotal role in HD pathogenesis. Epigenetic regulation of HD by miRNAs could provide a viable target for future therapeutics, particularly when one miRNA, such as miR-128a, regulates multiple genes within the HD signaling pathway.

## Methods

### HD monkeys and preparation of brain tissues

Brain tissues were collected from 11 monkeys (HD1-HD8 and C1-C3) (Additional file 1: Table S1). C2 and HD5 were miscarried at 3 months of gestation and C1, C3, HD1, HD2, HD3, HD4, and HD6 were miscarried at 4 months of gestation. HD7 and HD8 were delivered at full term and euthanized on the first day after birth because of severe involuntary movement and swallowing or respiratory difficulty, which was previously reported as rHD4 and rHD5, respectively [53]. C1 and HD4 were twins, and HD7 and HD8 were also twins. C1-C3 carried green fluorescent protein (GFP) transgene, and HD1-HD8 carried the *mHTT* transgene (exon 1 driven by human polyubiquitin promoter with expanded polyQ) and *GFP* gene [53]. Brain samples were prepared as soon as miscarried fetuses were retrieved or immediately after euthanasia. One hemisphere was post-fixed in 4% paraformaldehyde (PFA) and processed as described in the following section while the contralateral hemisphere was immediately dissected into small pieces (~0.5 cm^3^) according to distinct brain regions. The dissected brain regions were snap frozen in liquid nitrogen and stored at −80°C until analyzed. Sequence and copy analysis of all monkeys (Additional file 1: Table S1) was carried out according to previously published protocols [53].

### Immunocytochemistry

Post-mortem brain tissues were fixed in 4% paraformaldehyde overnight and then saturated in 30% sucrose solution at 4°C. The brain was then cryo-sectioned coronally to 50 μm using a cryostat and preserved in cryoprotectant solution at −20°C. Because most monkeys were miscarried and their brains were immature and soft, some of the monkey brains were not adequate for sectioning and subsequent immunohistochemical studies. Thus, only sections of frontal cortex from C1, C3, HD4, HD7 and HD8 were used for neuropathological study. For DAB immunostaining, sections were incubated with 0.3% hydrogen peroxide for 15 minutes, blocked for 1 hour at room temperature, and incubated with primary antibody (mEM48 1:1000, caspase-3 1:1000, or GFAP 1:1000) at 4°C overnight. After thorough wash with DPBS, brain sections were incubated with avidin–biotin using the Vectastain Elite ABC kit (Vector Laboratories), and immediately stained with 3, 3’-diaminobezidine (DAB; Vector Laboratories) for 30–40 seconds until optimal signal was reached. The sections immunostained with caspase-3 were restained in hematoxylin (Richard-Allan scientific) for nuclear staining. Brain sections were then mounted on slides and examined by Olympus BX51 microscope.

### Western blotting

Frontal cortex tissues from five individual monkeys (C1, C3, HD4, HD7 and HD8) were homogenized in RIPA buffer with protease cocktail inhibitor (Sigma). The tissue lysates were sonicated for 10 seconds and then centrifuged for 4 minutes at 1000 rpm at 4°C to pellet cellular debris. Protein concentrations were determined by protein assay. Equal amount (30–40 μg) of protein extract with loading dye was boiled for 5–10 minutes before loading into 9% (mEM8 and GFAP) polyacrylamide gels. After electrophoresis, proteins were transferred onto PVDF membrane (Bio-Rad) followed by blocking in 5% skim milk for 30 minutes. The membrane was incubated with the primary antibodies mEM48 (1:50 dilution) or GFAP (1:1000) overnight at 4°C, followed by the secondary peroxidase-conjugated antibodies for 1 h. Protein bands were detected with an Amersham ECL kit (PerkinElmer). Membrane was then labeled with γ-tubulin (1:2000) as internal control.

### Antibodies

Primary antibodies used in this study are as follows: 1) mouse monoclonal antibody (mEM48) against the N-terminal region of human HTT with expanded polyQ was a gift from Dr. XJ Li lab [54]; 2) rabbit antibody against the activated form of caspase-3 (cleaved caspase-3 (Asp 175) antibody) was from Cell Signaling; 3) mouse anti-Glial Fibrillary Acidic Protein (GFAP) monoclonal antibody was purchased from Millipore; 4) the mouse anti-gamma-tubulin antibody was purchased from Sigma. Secondary antibodies for immunohistochemistry were biotinylated goat anti-rabbit IgG (caspase-3 immunohistochemistry) or biotinylated horse anti-mouse IgG (mEM48 and GFAP immunohistochemistry) from Vector Labs (Burlingame, CA). Secondary antibodies for Western blotting were peroxidase-conjugated donkey anti-rabbit or anti-mouse IgG from Jackson Immunoresearch Labs.

### Qualitative analysis of caspase-3 and mEM48

Immunostained sections were examined using a Leica DMRB microscope, photographed at a magnification of 10–100 (camera: Leica DC500; Varshaw Scientific, Atlanta, GA, USA) and captured with computer software (SPOT Basic). The percentage of caspase-3 positive neurons was calculated based on number of positive cells per total nuclei. Five randomly captured images at 40× magnification were used to determine the number of caspase-3 positive and total number of nuclei for each monkey. Brain sections were labeled with mEM48 and used to determine the percentage of the number of EM48 (mHTT) positive cells with intranuclear inclusions. Eight randomly captured images at 20× magnification were used to determine the number of total number of EM48 positive cells with intranuclear inclusion for each monkey. For quantitation of protein expression by densitometry with Western blot, bands were captured and the intensity of positive bands was quantified with ChemiDox (BIORAD Inc.). All data are presented as mean ± standard error of the mean (SEM), and analyzed by one-way ANOVA using Graphpad Prism software, and followed by Newman-Keuls method for comparisons between groups.

### *mHTT* transgene characterization

The monkeys were genotyped and estimated copy number of the mHTT transgene were obtained following previous published protocols [53]. Additionally, we detected the mutant transgene by gel electrophoresis after primer-specific PCR amplification. The genomic DNA (gDNA) was extracted from frontal cortex tissue using the Wizard Genomic DNA Purification Kit from Promega following the manufacturer’s protocol. For genomic amplification of the *HTT* transgene, 60 ng of gDNA for the 8 HD transgenics and 3 GFP controls was analyzed by PCR using SsoFast EvaGreen Supermix from BioRad at a 60.5°C annealing temperature. Primers were designed to specifically amplify the junction of the human polyubiquitin C promoter and 5’ region of the exon1 of the *mHTT* transgene (see Additional file 2: Table S2). qPCR was performed using the CFX96 BioRad CyclerAll amplified products were analyzed by 1.5% agarose gel and subsequently sequenced for transgene verification.

### qPCR analysis of HTT mRNA in HD monkeys

Quantitation by qPCR was engaged to compare HTT mRNA levels between the HD and control monkeys. Total RNA from the cortical tissue was isolated using Trizol and subsequently treated with DNase to remove residual genomic DNA contamination. 200 ng of purified RNA was reverse-transcribed to cDNA (Applied Biosystems). To quantitate the mRNA levels of HTT, we custom designed Taqman assays (ABI) specific for the gene (see Additional file 2: Table S2). HTT transcript was amplified from the cDNA by qPCR using the Taqman assay with 1X final concentration of Taqman Universal PCR Master Mix (ABI). All data were normalized to 18S rRNA levels. The integrity of all RNA analyzed in this study was assessed by both a Bioanalyzer and RNA gel electrophoresis. All RNA was determined to be intact by the presence of distinct 28SRNA and 18SRNA ribosomal bands and a greater intensity of the 28SrRNA compared to the 18SrRNA (see Additional file 5 for RNA gel).

### miRNA array profiling

Total RNA was isolated from the frontal cortex of all 3 control and all 8 HD transgenic Rhesus monkeys using Trizol reagent (Invitrogen) according to manufacturer’s recommendations. RNA samples were sent to Ocean Ridge Biosciences for analysis using custom multi-species microarrays containing 496 probes covering 505 mature miRNAs (rhesus) present in the Sanger 15.0 miRBase database. The microarrays were produced by Microarrays, Inc. (Huntsville, Alabama) and consisted of epoxide glass substrates with each probe spotted in triplicate. Quality of each total RNA sample was assessed using UV spectrophotometry and agarose gel electrophoresis. Low molecular weight (LMW) RNA (~0-200 nucleotides) was purified from total RNA by size fractionation and 3’-end labeled with Oyster-550 fluorescent dye using the Flash Tag RNA Labeling Kit (Genisphere Inc., Hatfield, PA). Labeled LMW RNA samples were hybridized to the arrays according to conditions recommended in the Flash Tag RNA labeling Kit manual. The microarrays were scanned on an Axon Genepix 4000B scanner and data were extracted from images using GenePix V4.1 software.

### miRNA qPCR quantitation of in monkey and human brain

Dysregulated miRNAs which had predicted targets with significant HD pathway association, as determined by Ingenuity Pathways Analysis (p-value < 0.05), were subjected to further validation by qPCR. Taqman miRNA assays (ABI) were used to quantify levels of the selected candidates. The assay utilized miRNA-specific primers for both cDNA synthesis and qPCR, and all reactions were normalized to RNU48 as a small RNA endogenous control. Additionally, total RNA was prepared by Trizol-chloroform extractions from human samples for quantitation of miR-128a by qPCR. Human HD brain tissues were provided by Emory Alzheimer’s Disease Research Center and the Emory Neuroscience NINDS Core Facilities (ENNCF) Neuropathology Core Service at Emory University. The use of human tissues was followed and compiled with NIH guideline. All miR-128a qPCR values in the human tissues were normalized to the small RNA endogenous control, RNU6B.

### TargetScan and Ingenuity pathway analysis of miRNA candidates

The predicted mRNA targets for all of the 11 miRNAs significantly dysregulated with HD by microarray (P value < 0.05) in our study were identified using the TargetScan prediction program. The predicted mRNA targets for each were then subjected to pathway analysis using the Ingenuity software to identify potential association with the HD signaling pathway (P value < 0.05).

### Luciferase reporter assays

3’-UTR sequence information was obtained for the rhesus genes *HIP-1, HTT, SP-1* and *GRM5* using TargetScan (Release 5.2: June 2012), UCSC Genome Browser, and NCBI databases. Sense and anti-sense oligos spanning the predicted miR-128a binding sites in each gene’s 3’UTR were custom ordered (Eurofin Operon) (see Additional file 2: Table S2 for oligo sequences). Additionally, sense and anti-sense oligos with point mutations in 4 of the 6 nucleotides of the seed region for the miR-128a binding sites were generated for use as mutant (MUT) controls. Sense and anti-sense strands for each site were annealed and ligated into the pmirGLO Dual-Luciferase miRNA Target Expression Vector (Promega). Constructs were transformed into High Efficiency JM109 Competent Cells (Promega). Annealing, ligation, and transformations were performed according to manufacturer’s recommendations. Selected clones were mini-prepped using a QIAGEN Plasmid mini-prep kit, and inserts/sequences were confirmed using restriction digests and sequencing.

HEK-293FT cells were used for 3’UTR Luciferase reporter transfections. Lipofectamine 2000 (Invitrogen) was used to transfect plasmids/miR-128a into 293FT cells according to manufacturer’s recommendations in 96-well plates (coated with 0.1% gelatin, 14,000 cells/well). Each well was transfected with 40 ng of plasmid alone (controls), or in combination with 10 nM pre-miR-128a (Ambion, catalog AM17100) or Negative Control #2 (Ambion). Following transfection, cells were grown for 24 hours at 37ºC and luciferase levels were quantified using the Dual-Glo Luciferase Assay (Promega) according to vendor instructions; measurements were read using a Synergy H4 plate reader (BioTek Instruments, Inc.). All Firefly luciferase data were normalized to Renilla luciferase levels; experiments were run in quadruplicate and individual experiments were replicated at least once.

## Competing interests

The authors declare that they have no competing interests.

## Authors’ contributions

JK prepared brain tissues for miRNA array, validated candidates, performed data analysis and wrote the manuscript. YX performed neuropathological studies. JK and MSP designed and performed luciferase assays. DZ performed cell culture and protein analysis. AWSC planned, designed, oversaw all studies, performed data analysis and interpretation, wrote and approved the paper. All authors read and approved the final manuscript.

## Supplementary Material

Additional file 1: Table S1 Transgenic monkey information. Click here for file

Additional file 2: Table S2 Primer and oligonucleotide sequences. Click here for file

Additional file 3 miRNA array data. Click here for file

Additional file 4: Table S3 Ingenuity-miRNA target associations. Click here for file

Additional file 5 Evaluation of RNA integrity. Click here for file
