# Supplementary material for: microRNA-128a dysregulation in transgenic Huntington’s disease monkeys
Source: Mol Brain. 2014 Jun 13;7:46. doi: 10.1186/1756-6606-7-46 (PMC4065582; doi:10.1186/1756-6606-7-46)

Additional File 5: Figure S1 RNA integrity gel

- Note, equal volumes of RNA were loaded and were not loaded on the gel according to equal concentration

| **Lane** | **Sample ID** |
| --- | --- |
| **1** | HD1 |
| **2** | rHD2 |
| **3** | HD3 |
| **4** | C1 |
| **5** | rHD4 |
| **6** | C2 |
| **7** | HD5 |
| **8** | HD6 |
| **9** | C3 |
| **10** | sample from monkey not analyzed in this study to use as another RNA quality control |
| **11** | HD7 |
| **12** | HD8 |
| **L3** | **Transsteps RNA ladder** |


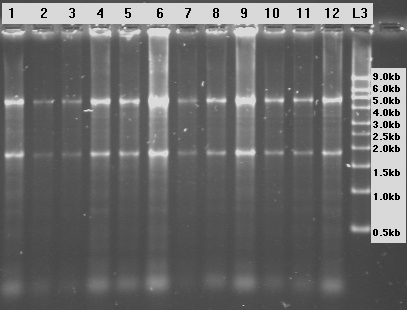

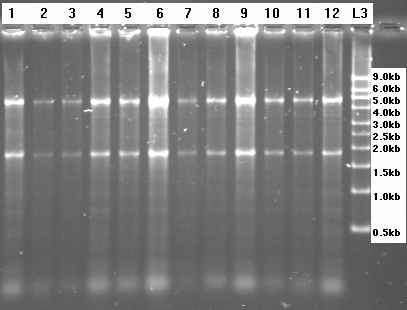

Supplement: Additional file 5 — Evaluation of RNA integrity. [file 1756-6606-7-46-S5.docx]
